# Canola Proteins for Human Consumption: Extraction, Profile, and Functional Properties

**DOI:** 10.1111/j.1750-3841.2010.01930.x

**Published:** 2011-01

**Authors:** Siong H Tan, Rodney J Mailer, Christopher L Blanchard, Samson O Agboola

**Keywords:** brassica, canola, extraction, functional properties, protein isolate

## Abstract

Canola protein isolate has been suggested as an alternative to other proteins for human food use due to a balanced amino acid profile and potential functional properties such as emulsifying, foaming, and gelling abilities. This is, therefore, a review of the studies on the utilization of canola protein in human food, comprising the extraction processes for protein isolates and fractions, the molecular character of the extracted proteins, as well as their food functional properties. A majority of studies were based on proteins extracted from the meal using alkaline solution, presumably due to its high nitrogen yield, followed by those utilizing salt extraction combined with ultrafiltration. Characteristics of canola and its predecessor rapeseed protein fractions such as nitrogen yield, molecular weight profile, isoelectric point, solubility, and thermal properties have been reported and were found to be largely related to the extraction methods. However, very little research has been carried out on the hydrophobicity and structure profiles of the protein extracts that are highly relevant to a proper understanding of food functional properties. Alkaline extracts were generally not very suitable as functional ingredients and contradictory results about many of the measured properties of canola proteins, especially their emulsification tendencies, have also been documented. Further research into improved extraction methods is recommended, as is a more systematic approach to the measurement of desired food functional properties for valid comparison between studies.

## Introduction

The name canola was introduced in Canada in 1979 that specifically denotes rapeseed varieties that produce oil having less than 2% erucic acid and less than 30 μmol/g meal of total glucosinolates (Canola Council of Canada 1990). These Brassica varieties are sources for some of the healthiest vegetable oils for human consumption (Downey and Bell 1990), as well as a potential source for manufacturing a wide variety of environment-friendly products such as biodiesel and bioplastics (Wu and Muir 2008). Canola seed typically contains over 40% oil (Kimber and McGregor 1995) and in Australia, the average oil content for the 2008 canola harvest was 41.8% (Seberry and others 2008).

The annual worldwide growth of canola production has been phenomenal and is predicted to exceed 15 million tonnes by 2015 (Canola Council of Canada 2009). In Australia, the world's 2nd-largest exporter of canola seed after Canada, canola is also the major oilseed crop with production being maintained at 1.5 million tonnes and contributing up to 96% of the total oilseeds production in Australia since 2000. According to Australian Oilseeds Federation (2009), future prospects for the Australian oilseeds (canola) industry are excellent and the Australian oilseeds industry is expected to grow to a value of $3.3 billion by the end of 2010. The growing demand for canola oil worldwide implies that more meal will be produced as a result of the increased oil extraction. This demonstrates the need for a better understanding and knowledge of canola proteins, the major constituents in the meal.

The protein rich meal, which is left behind after the oil has been removed from the seed, is currently used as a protein source in livestock and aquaculture industries (Uruakpa and Arntfield 2005a; Canola Council of Canada 2009). Other than as animal feed, canola meal has been suggested as a potential alternative to plant proteins for human consumption (Uppstrom 1995). It has been found to have high biological value (Campbell and others 1981) and known for its well-balanced amino acid composition (Sosulski 1983; Pastuszewska and others 2000). There are also indications that canola proteins have good technologically functional properties (Aluko and McIntosh 2001; Yoshie-Stark and others 2008). All these suggest that canola meal is a valuable source for the isolation of high-quality protein for utilization in the food processing industry, as a good alternative to soybean derivatives and other plant and animal products.

This article provides a review of available research on defatted canola meal proteins and their potential use in human food manufacture. As indicated, rapeseed and canola seed are different only in regard to their erucic acid content in the extracted oil and total glucosinolates level in the meal. Thus, in this review, due to its focus on protein, the term “rapeseed” and “canola seed” are used interchangeably. In section 2, we will review the factors affecting the potential use of canola meal and proteins in human food and issues that may need to be considered before this can be fully realized. Section 3 provides an overview for the extraction of canola proteins using different methods. In section 4, we review the profile and characteristics of canola proteins focusing on properties such as molecular size, protein structure, isoelectric point (pI), solubility, hydrophobicity, thermal properties, as well as details of their amino acid composition. Canola proteins’ food functional properties, especially emulsifying, foaming, and gelling abilities, are covered in section 5. This review is concluded with comments about the future prospects for canola protein utilization in foods and our recommendations for further studies.

## Factors Affecting the Utilization of Canola Meal Protein in Human Food Manufacture

### Antinutritional factors in canola meal

Antinutritional factors in the oil free canola meal are the major obstacle for its use in human food manufacture. Canola meal contains glucosinolates, phenolics, phytates, and a high amount of fiber that make it problematic for food use (Wu and Muir 2008; Yoshie-Stark and others 2008). The impact of these components leads to unacceptable properties of canola meal that include relatively inferior physicochemical properties, poor digestibility, objectionable color, and bad taste (Wu and Muir 2008).

Phenolic acid esters are considered as principal antinutritive factors in canola seeds (Sosulski 1979; Ismail and others 1981). Their concentration has been reported to be about 30 times higher than those in soybean (Kozlowska and others 1990; Shahidi and Naczk 1992). Specifically, the predominant phenolic compounds in seeds of oilseed rape are sinapate esters with sinapoylcholine (sinapine) being the most prominent one, followed by sinapoylglucose. Sinapate esters cause a dark color and bitter taste in rapeseed meal and extracted protein products (Zum Felde and others 2007). Besides, sinapate esters have negative effects on the digestibility of rapeseed meal. During rapeseed oil processing, sinapine may form complexes with protein through oxidation that then decrease the digestibility of rapeseed meal (Kozlowska and others 1990; Shahidi and Naczk 1992). Genetic variation determines the sinapate ester content in rapeseed meal. Velasco and Mollers (1998), in their study into 1361 rapeseed samples, reported a range of sinapate ester contents from 5 to 17.7 g/kg seeds of *Brassica napus*.

The glucosinolates level in canola meal is relatively high at 18 to 30 μmol/g meal and has been shown to have antinutritional or toxic effects in animal studies (Sorensen 1990). Interestingly, a lower level of glucosinolates content has been reported to have positive effect on health. Song and Thornalley (2007) found that glucosinolates level of 0.61 μmol/g in broccoli can be linked to a reduced cancer risk. Nevertheless, it is a fundamental task to reduce the glucosinolates level so that the proteins extracted from canola meal are fit for human consumption.

Phytic acid is another antinutritional factor in canola meal, typically existing as mixed salts (phytates) of Ca, Mg, and K (Mills and Chong 1977; Yiu and others 1982). This is mainly due to the fact that the molecule is negatively charged at normal pH; therefore, it is very reactive with cations such as minerals (Murthy and Rao 1986; Thompson and Serraino 1986). The formation of phytic acid-mineral complexes thus decreases the availability of minerals. Phytate levels of 2.0% to 5.0% have been reported for the defatted meal, and up to 9.8% for the protein isolates and concentrates depending on the method of protein isolation (Uppstrom and Svensson 1980; Thompson 1990). Other than binding with minerals, phytic acid also binds to proteins, reducing the protein digestibility, and amino acid availability (Thompson 1990). Furthermore, phytic acid has been shown to reduce amylase activity, thus reducing starch digestion and absorption (Yoon and others 1983). However, this is an issue only if the canola meal is incorporated in a mixed diet, since canola seed does not contain starch. These anitinutritional factors thus make it almost impractical to use canola/rapeseed protein in any meaningful way for human food. This also explains why the current use of rapeseed meals is generally restricted to only animal feed and fertilizer. Furthermore, rapeseed meals may also induce allergy in hypersensitive individuals (Monsalve and others 1997).

### Removal of antinutritional factors

Many studies have been carried out with the objective of removing or reducing antinutritional factors in rapeseed and canola. Naczk and others (1985) reported a 2 phase solvent extraction system to produce canola meal with glucosinolate content decreased to trace levels. A protein extraction method, which is based on the formation of protein micellar mass (PMM), has proven to be efficient in removing glucosinolates with minimal loss of proteins (Tzeng and others 1990a), with the reduction in glucosinolate level being associated with the ultrafiltration step as the toxic compounds have significantly lower molecular weights than rapeseed proteins (Ser and others 2008).

The effect of processing on the antinutritional factors of rapeseed has also been studied. Mansour and others (1993) reported a reduction of up to 94% of glucosinolates, 43% of phytic acid, and 67% of tannic acid in the canola meal tested when subjected to heat treatment. Jensen and others (1995) have reported similar findings that glucosinolates were destroyed by high temperature, thus improving the canola meal flavor and palatability. Application of organic solvents, such as ethanol, methanol, and acetone, is another efficient way to remove glucosinolates from canola meal (Mawson and others 1995). Jensen and others (1990) also reported the use of enzymes, such as pectinase, protease, and hemicellulase, in reducing the glucosinolates content.

Transgenic approaches were being taken as an alternative in dealing with the antinutritional factors that resulted in rapeseed lines with reduced sinapate ester contents (Nair and others 2000; Husken and others 2005). However, because of inefficient analytical methods and other complications, breeding programmes aimed at developing rapeseed cultivars with low sinapate content have not been successful (Zum Felde and others 2007).

Although canola meal and associated proteins have been acknowledged as having profile and quality that made them suitable for human consumption, it is equally important to process them in such a way that minimize the level of antinutritional factors. Therefore, any new processing method for the protein isolates must establish a clear pathway for their incorporation into human foods without significant effects on sensory and nutritional qualities.

## Canola Protein Extraction

### Preparation of seed meals

Canola seeds are typically crushed or ground to aid the separation and defatting process, usually in a Sohxlet apparatus. Removal of fat from the crushed canola seed is normally carried out using hexane as solvent (Tzeng and others 1988a; Wu and Muir 2008). The defatted meal is usually dried at room temperature in a fume hood (Aluko and McIntosh 2001; Ghodsvali and others 2005) or under vacuum in an oven at 40 °C (Tzeng and others 1990a). The dried and defatted meal may then be ground to pass through 40-mesh (Aluko and McIntosh 2001) or 60-mesh (Wu and Muir 2008) screen in order to assure thorough interaction of the meal with chemicals during the protein extraction process. An alternative method for preparing defatted meals was reported by Tzeng and others (1988a, 1990a), where the canola seeds were ground to slurry using an orbital mill in the presence of hexane.

### Protein extraction by alkaline solution

**Figure 1:**
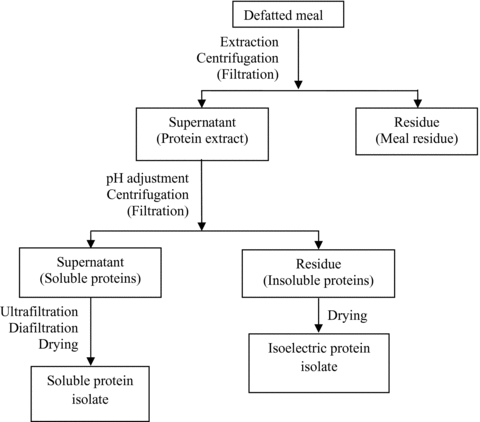
Schematic representation for alkaline extraction of canola meal protein isolates.

Alkaline extraction with sodium hydroxide (NaOH) solution followed by precipitation with dilute acid is the most typical procedure used in preparation of canola protein isolates (CPIs) (Klockeman and others 1997; Aluko and McIntosh 2001). The reported extraction procedures, however, had slight differences in pH of extraction, concentrations of NaOH used, centrifugation and filtration settings, type of acid, and pH for protein precipitation. Generally, the alkaline solution was first added to the defatted canola meal and stirred or shaken for a given period of time to solubilize the proteins. The mixture was then centrifuged, and the pH of supernatant was adjusted by dilute acid to precipitate the proteins. Precipitated protein was then separated by centrifugation and the precipitate was freeze-dried (Figure 1). Comparison of alkaline extraction procedures used in different studies is shown in Table 1.

**Table 1:** Summary of alkaline extraction procedures for canola meal proteins

Procedure	Aluko and McIntosh (2001), Aluko and others (2005)	Pedroche and others (2004)	Klockeman and others (1997)	Ghodsvali and others (2005)	Tzeng and others (1988a, 1988b)	Tzeng and others (1990a)
Alkali extraction	10 volumes (w/v) of solution of 0.1 M NaOH 20 min, stirred at 23 °C	10 volumes (w/v) of solution of 0.2% NaOH, 1 h, pH 10, 11, 12, twice	5% (w/v) extraction with 0.4% w/v NaOH, T_room_, orbital shaker 180 to 200 rpm, 60 min	5% NaOH, R = 18, pH 9.5, 10, 10.5, 11, 11.5, 12	1.0% w/v aqueous SHMP, pH 9, (or NaOH pH 11), *R*= 18, 30 min	NaOH solution, *R*= 18, pH 11 or 12 1% Na_2_SO_3_ added
1st centrifugation	10000 ×*g*, 30 min, 8 °C	8000 rpm, 25 min	3000 ×*g*, 20 min, 5 to 10 °C	5000 rpm, 15 min	4080 ×*g*, 10 min, 5 °C	4080 ×*g*, 10 min, 5 °C
Filtration	Yes	No	No	Yes	Yes	Yes
pH adjustment by acid	pH 4.0, by 0.1 M HCl	pH 2.5 to 6.0 in 0.5 increments by 0.5 N HCl	pH 3.5, by acetic acid	pH 3.5 to 7.5 in 0.5 increments by 6 N HCl	pH 3.5, by 6 N HCl solution	pH 3.5, by 6 N HCl solution
2nd centrifugation	10000 ×*g*, 30 min, 8 °C	8000 rpm, 25 min	3000 ×*g*, 20 min, 5 to 10 °C	5000 rpm, 20 min	Centrifuged, but no details given	Centrifuged, but no details given
Washing	Washed (200 volumes of Mili-Q water) to remove salt	Precipitates were mixed	Precipitate was washed by distilled deionized water	Filtration, washed with distilled water, *R*= 10, repeated twice	Filtration, washed (10 volumes of acidic water pH 3.5), shaking, 2 h	Filtration, washed (10 volumes of acidic water, pH 3.5)
	Centrifuged 10000 ×*g*, 30 min, 8 °C		Centrifuged 3000 ×*g*, 20 min 5 to 10 °C, repeated 3 times		Centrifuged, repeated 3 times.	
Drying method	Freeze-dried	Freeze-dried	Freeze-dried	Freeze-dried	Freeze-dried	Freeze-dried
Collecting soluble protein isolate	No	No	No	Yes	Yes	Yes

SHMP = sodium hexametaphosphate. R = solvent to meal ratio. CF = concentration factor, for example, 10, meaning 100 g of protein solution was concentrated to 10 g by ultrafiltration. DV = diavolume, for example, 5 meaning 100 g of sample was diafiltered with 500 g water.

The use of alkali, as shown by Sosulski (1983) and Mieth and others (1983), produce strong conditions (pH 11 to 12) that were necessary to obtain high nitrogen extraction yield and a high protein extraction rate from canola meal. Tzeng and others (1988a) maintained the pH of the solution by the addition of 50% w/w NaOH solution. Besides, 10% sodium sulphite (Na_2_SO_3_) was added during the extraction process (Tzeng and others 1990a) to inhibit oxidation of phenolic compounds, thus preventing the possible reaction between proteins and phenolic compounds. The protein isolates produced thus had a light ivory color. Addition of Na_2_SO_3_ has, however, not been observed in most other studies.

The use of sodium hexametaphosphate (SHMP) as an alternative extraction solution to NaOH has been reported. Thompson and others (1976) successfully developed a process using 2% aqueous SHMP solution in rapeseed protein extraction. Tzeng and others (1988b) also reported that SHMP is an effective extraction agent for rapeseed protein. Further study by Tzeng and others (1988a) showed that extraction by SHMP, if compared to NaOH, produced isolates of better color and taste. The yield, however, was only 7% of the meal solids, accounting for 18% of the nitrogen in the meal. This low yield could possibly explain why in the majority of the canola protein studies reported in recent years; the extractions were carried out by using NaOH instead of SHMP.

The centrifugation step was conducted to separate the meal residue from the extracted proteins in alkali solution. Filtration after the initial centrifugation was reported in majority of the extraction procedure (Tzeng and others 1990a; Aluko and others 2005). This extra step ensures no contamination of supernatant from the precipitates. Precipitates at this stage can be discarded or washed with alkali solution of the same pH as the extraction solution and oven dried to collect the meal residue (Ghodsvali and others 2005). The adjustment of the pH of the extract's supernatant to the pI is normally carried out by using dilute acid solutions. Aluko and McIntosh (2001) and Aluko and others (2005) suggested adjusting the pH to 4 using 0.1 M HCl. Adjustment of pH to 3.5 has also been reported by using acetic acid (Klockeman and others 1997) or hydrochloric acid (Tzeng and others 1990a). Comparatively, Ghodsvali and others (2005), in their study on extraction of protein from 3 different canola varieties, adjusted the extracted proteins from pH 3.5 to 7.5 in increments of 0.5 pH units and found the range of pH 4.5 to 5.5 as the optimum pH for protein precipitation. Lonnerdal and Janson (1972) suggested that a large proportion of canola proteins (20% to 40%) have pIs close to pH 11, while the other proteins have pIs spread out in the interval of pH 4 to 8.

In some studies, such as work carried out by Pedroche and others (2004), more than one pI was reported. At both pH 3.5 and 5, different protein fractions extracted from *Brassica carinata* defatted meal achieved their lowest solubility (Pedroche and others 2004). Thus, during the extraction, the extract was adjusted to pH 5, centrifuged, and a precipitate was collected. The pH of the supernatant was then further adjusted to pH 3.5, and centrifuged. This 2nd fraction was collected and mixed with the 1st fraction before freeze-drying. El Nockrashy and others (1977) in their studies on *B. napus* proteins, also reported similar procedure. The protein shows 2 pIs at pH 3.6 and 6.0, at which 57% to 65% of the total nitrogen, corresponding to 70% to 80% of meal protein in the extract was precipitated.

A 2nd centrifugation step was usually conducted to separate the acid precipitated (insoluble) proteins from the soluble proteins (supernatant), followed by washing with distilled deionized water (Klockeman and others 1997) or Mili-Q water (Aluko and McIntosh 2001; Aluko and others 2005). The washed precipitate was then freeze-dried to produce the isoelectric protein isolate.

Addition of CaCl_2_ prior to (Tzeng and others 1988a, 1990a; Ghodsvali and others 2005) or after (Aluko and McIntosh 2001; Aluko and others 2005) the pH adjustment for isoelectric protein precipitation has been reported to produce low phytate calcium-precipitated protein isolates. Tzeng and others (1990a) found that addition of 0.15 M CaCl_2_ produced a phytate free soluble protein isolate. The percentage of soluble protein also increased to approximately 80% of the total yield, but at the same time, precipitated protein was decreased to about 20%. This high yield is due to the “salting in” effect, as demonstrated clearly by Tzeng and others (1990a) in their study. Diosady and others (1989) showed that an approximation of 0.05 M CaCl_2_ was the optimum concentration suggested in terms of protein recovery and phytate content. However, there was little information in literature regarding the properties of calcium-precipitated protein isolates in comparison to those of acid-precipitated protein isolates.

Soluble protein extracts were collected following an additional procedure by Ghodsvali and others (2005). All washing liquids and supernatant from the 2nd centrifugation were combined and ultrafiltered, at a concentration factor (CF) of 10, followed by diafiltration at a diavolume (DV) of 5. The retentate was freeze-dried to produce the soluble protein isolate. Ultrafiltration process was able to remove water, glucosinolates, nonprotein nitrogen, and nitrogen free material, while at the same time concentrating the protein before the drying process (Diosady and others 1984).

Current literature shows that the protein content of isolates prepared by alkaline extraction was mostly in the range of 70% to 90% (Aluko and McIntosh 2001; Ghodsvali and others 2005), although isolates with protein content more than 90% have also been reported (Tzeng and others 1988a; Pedroche and others 2004).

### Protein extraction by PMM method

PMM method has been developed as an alternative process for extracting canola meal proteins. This method reduced the concentration of problematic antinutritional or toxic factors, including the glucosinolates and their degradation products (Burgess 1991; Ismond and Welsh 1992). One of the most recent studies on protein isolates by using PMM method was reported by Ser and others (2008) who adapted the PMM method of Ismond and Welsh (1992).

**Figure 2:**
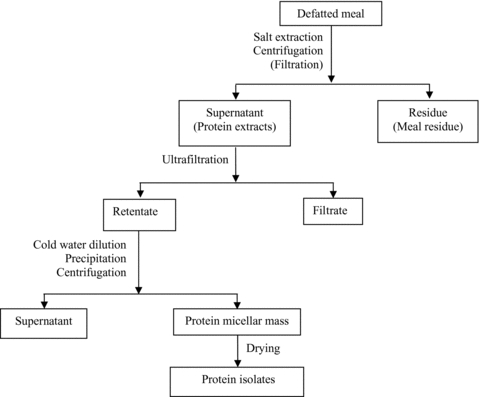
A schematic of the protein micellar mass method used for extracting canola meal protein isolates. Source: Ser and others (2008).

PMM method is made up of 4 main steps that consist of extraction, ultrafiltration, dilution, and precipitation. The defatted meal was first extracted by sodium chloride (NaCl) solution, followed by ultrafiltration process to concentrate and purify the proteins. This ultrafiltration step has proven to be efficient in removing glucosinolates with minimal loss of proteins (Tzeng and others 1990b). The retentate was then diluted with cold water to reduce the ionic strength of the concentrated protein and promote precipitation. Burgess (1991) suggested a dilution factor of 1 to 6 to precipitate the purified salt extracted canola protein effectively through the formation of protein micelles. The protein micelles were then separated from the water through centrifugation. Precipitates were collected and freeze-dried (Figure 2).

A similar extraction process had been reported by Owen and others (1971), Raab and Schwenke (1984), and more recently by Wu and Muir (2008). However, in comparison to the alkaline extraction method, there is not much literature on PMM for protein extraction. This could possibly be due to the poorer overall protein yield (71.3% to 78.5%) as reported by Owen and others (1971) and Ismond and Welsh (1992) in comparison to protein yield from alkaline extracts.

### Preparation of specific protein fractions

As noted, the majority of studies on the extraction of canola meal protein isolates were carried out by using alkaline solution with a few using either the PMM method or salting out with NaCl. Furthermore, canola protein was extracted as a whole rather than as specific protein fractions. Osborne (1897) however suggested categorizing proteins based on their solubility in water (albumins), salt solution (globulins), alkaline solution (glutelins), and alcohol (prolamins). From the alkaline extraction process discussed above, we could, theoretically, refer to the alkali-extracted protein as glutelins based on the Osborne scheme. However, because NaOH is a strong alkali, and there was no prior extraction of canola meal proteins either by water or salt solution before the alkaline extraction, it is safe to conclude that the proteins extracted were a combination of some or all of the Osborne fractions. The results of electrophoretic analysis by Aluko and McIntosh (2001) confirmed the 12S globulins as being part of the proteins obtained from the alkaline extraction, demonstrating that the globulins, which are soluble in salt solution, can also be extracted by the strong alkaline solution.

Bhatty and others (1968) were the first to study Osborne fractions of rapeseed protein and isolated one of the protein fractions (globulins) from oil free rapeseed meal by using 10% NaCl, followed by precipitation by dialysis and chromatographic purification. The full procedure in extracting all 4 Osborne fractions was described by Betschart and others (1977) based on the modification of the classical procedure of Osborne and Mendel (1914). Samples were thrice extracted by shaking for 2 h in distilled water (solvent to meal ratio, 20:1); extracts were pooled and centrifuged at 3000 g for 20 min. The extracted albumin fractions (supernatant) were filtered through Whatman nr 1 paper and dialyzed at 2 °C through a cellulose acetate membrane against 20 volumes of distilled water with 2 changes for 24 h. Extracts were then freeze-dried at shelf temperatures of 15 °C and milled to pass through a 20-mesh screen. The residue from the centrifuge was similarly extracted with 5% NaCl, then 60% (v/v) ethanol, and finally by 0.4% NaOH to obtain globulins, prolamins, and glutelins, respectively.

Uppstrom (1995) found that majority of the rapeseed proteins are globulins, albumins, and oleosins. This is consistent with the data provided by Salunkhe and others (1992) based on the protein fractionation studies. Canola protein was made up of approximately 70% of salt soluble globulins, up to 20% of alcohol soluble prolamins, and 10% to 15% water-soluble albumins. Prolamins in rapeseed exist exclusively as oleosin, the structural proteins associated to the oil bodies (Mieth and others 1983). Klockeman and others (1997), however, reported that the isolated canola proteins were primarily glutelins and globulins.

CPIs were prepared mostly by direct alkaline extraction in comparison to fractionation with different solvents (Osborne method) presumably due to the high nitrogen yield of the extracts. However, based on the studies conducted on Osborne fractions of canola and other plant proteins, this method could be a better alternative for extracting proteins with particular profiles and characteristics in order to maximize their food processing functionalities.

## Protein Profiles and Characteristics

### Amino acid composition

The quality of proteins is very much determined by the amino acid composition, as amino acids are fundamental building blocks of protein. Amino acid composition of canola meal and its CPIs are shown in Table 2. Amino acid compositions of soy protein isolates (SPIs) and casein are also included for comparison, since they are considered as good sources of amino acid nutrition for infants and children by international standards (FAO/WHO/UNU 1985).

**Table 2:** Amino acid composition (g/100 g protein) for canola meal, extracted isoelectric protein, and soluble protein

References			Slominski and others (1999)					Tzeng and others (1988a, 1988b)	Tzeng and others (1988a, 1988b)					Wang and others (2008)	Wang and others (1999)	FAO/WHO/UNU (1985)			
								
			Meal						Isoelectric protein			Soluble protein		Soy protein isolate (SPI)	Casein	Infant^C^	Preschool child, 2 to 5 y	Child, 10 to 12 y	Adult
											
									SHMP extract	NaOH extract	SHMP extract	NaOH extract	SHMP extract						
Amino acid/symbol			Commercial meal “yellow”	Commercial meal “brown”	*B. rapa*“yellow” cv. Parkland	*B. napus*“brown” cv. Excel	*B. napus*“yellow” cv.Y1016	Canola *B. napus*, cv. Altex	Canola, *B. napus*, cv. Altex	Canola, *B. napus*, Altex cultivar		Canola, *B. napus*, Altex cultivar							
Essential	Arg	R	5.51	5.5	5.61	5.96	5.71	7.28	6.77	7.66	7.17	7.28	6.59	7.35	3.30				
	His	H	2.95	3	3.42	3.46	2.65	3.18	3.15	3.17	3.14	3.47	3.38	2.81	2.70	2.6	1.9	1.9	1.6
	Ile	I	4.05	4.11	3.96	4.2	3.99	4.47	3.87	5.18	4.42	4.01	3.90	4.35	4.90	4.6	2.8	2.8	1.3
	Leu	L	6.79	6.72	6.90	6.96	6.80	7.47	7.55	9.26	8.42	7.73	7.74	6.79	8.40	9.3	6.6	4.4	1.9
	Lys	K	5.61	5.53	5.71	5.75	5.54	6.60	6.34	5.62	5.04	5.94	5.7	5.23	7.10	6.6	5.8	4.4	1.6
	Met	M	1.9	1.92	2.07	1.93	2.04	2.24	2.18	2.6	2.18	2.35	2.23	0.92	2.60				
	Phe	F	5.89	5.65	5.95	5.80	5.55	4.67	4.72	5.13	5.28	4.06	4.32	5.14	4.50				
	Thr	T	4.01	3.82	4.10	3.99	3.81	4.81	4.49	5.30	4.52	4.13	4.00	3.98	3.70	4.3	3.4	2.8	0.9
	Val	V	4.88	4.86	4.85	5.11	4.93	5.65	4.92	5.85	5.46	4.80	4.90	4.28	6.00	5.5	3.5	2.5	1.3
	Try	W	N/A	N/A	N/A	N/A	N/A	N/A	N/A	N/A	N/A	N/A	N/A	N/A	N/A	1.7	1.1	0.9	0.5
Nonessential	Ala	A	4.33	4.32	4.38	4.44	4.36	4.53	4.15	5.14	4.63	4.34	3.91	3.72	2.70				
	Asp^A^	D	6.95	6.27	7.18	7.03	6.71	7.79	7.24	9.41	9.15	6.52	5.77	11.47	6.30				
	Cys	C	2.17	2.61	2.22	2.39	2.50	2.08	3.56	0.39	1.68	1.07	5.85	0.05	0.04				
	Glu^B^	E	17.07	17.48	17.27	17.95	17.39	20.81	23.21	17.27	20.19	26.17	24.17	20.67	19.00				
	Gly	G	4.87	4.59	4.98	4.82	4.75	4.60	4.32	5.05	4.94	4.64	4.27	3.74	1.60				
	Pro	P	6.16	6.57	6.29	6.15	6.47	6.22	6.06	4.32	5.92	6.95	6.96	5.13	N/A				
	Ser	S	4.78	4.69	4.87	4.89	4.75	4.41	4.40	4.74	4.27	4.09	3.71	5.32	4.60				
	Tyr	Y	3.04	2.98	3.13	3.04	3.04	3.19	3.07	3.93	3.60	2.44	2.6	3.61	5.50				
lys/arg			1.02	1.01	1.02	0.96	0.97	0.91	0.94	0.73	0.70	0.82	0.86	0.71	2.15				
Sulphur containing amino acid (met + cys)			4.07	4.53	4.29	4.32	4.54	4.32	5.74	2.99	3.86	3.42	8.08	0.97	2.64	4.2	2.5	2.2	1.7
Aromatic amino acid (phe + tyr)			8.93	8.63	9.08	8.84	8.59	7.86	7.79	9.06	8.88	6.50	6.92	8.75	10.00	7.2	6.3	2.2	1.9
Total essential amino acid			41.59	41.11	42.57	43.16	41.02	46.37	43.99	49.77	45.63	43.77	42.76	40.85	43.20				
Total amino acid			90.96	90.62	92.89	93.87	90.99	100	100	100	100	99.99	100	94.56	82.94				
Proportion of total essential			0.46	0.45	0.46	0.46	0.45	0.46	0.44	0.50	0.46	0.44	0.43	0.43	0.52				
amino acid to the total																			
amino acid																			

A Aspartate + asparagine.

B Glutamate + glutamine.

C Based on average amino acid composition of human milk.

N/A = not available.

Rapeseed protein tended to contain less lysine than soybean protein (Bell 1995). As shown in Table 2, lysine content of CPI (*B. napus*, cv. Altex) was in a range of 5.04% to 6.34% depending on the methods of extraction. NaOH extraction produced CPI that contained lysine. This was higher than the lysine content of CPI produced by extraction with SHMP (Tzeng and others 1988a). Comparatively, Pedroche and others (2004), in their study of amino acid profile of *B. carinata* proteins reported a lower content of lysine, with the isolate extracted by NaOH at pH 10, 11, and 12 giving lysine contents of 3.8%, 3.3%, and 4.5%, respectively.

Table 2 also shows that canola protein from either NaOH or SHMP extractions have at least 2.99% of sulphur-containing amino acids (methionine + cysteine). This amount exceeded the requirement of FAO/WHO/UNU (1985) for children and adults. It is also closer to requirements for infants in comparison to SPIs or casein, which were only 0.97% and 2.6%, respectively (Wang and others 1999, 2008). Ohlson and Anjou (1979) found that the content of sulphur-containing amino acids in rapeseed protein was higher than in any other vegetable protein. A number of studies have shown that there is only 1.39% in hempseed protein isolates, 0.92% in SPI (Wang and others 2008), 2.10% in chickpea protein isolates (Sanchez-Vioque and others 1999), and 1.31% in flaxseed whole extracts (Chung and others 2005).

CPI contains a substantial amount of threonine (4.49% to 5.30%), which is higher than threonine content in both SPI (3.98%) and casein (3.70%). It also exceeded the requirements set by FAO/WHO/UNU (1985) for all groups including infants. CPI contains 7.66% arginine, comparable to SPI, and more than twice the amount reported for casein. Another Brassica oilseed type-*B. carinata* has even higher content of arginine as reported by Pedroche and others (2004), ranging from 8.30% to 9.10%, depending the pH of extraction.

The lysine/arginine ratio is a determinant of the cholesterolaemic and antherogenic effects of a protein (Czarnecki and Kritchevsky 1992). Depending on the extraction method, lysine/arginine ratio for *B. napus* (cv. Altex) were in the range of 0.7 to 0.9, much lower than lysine/arginine ratio for casein protein (2.2), suggesting that CPI is less lipidemic and atherogenic than casein protein.

Glutamine is the most abundant amino acid in CPI, SPI, and casein. Depending on the extraction method, CPI contains 17.27% to 23.21% of glutamine, comparable to the glutamine content in SPI (20.67%) and casein (19.00%). Histidine content of CPI was higher (3.14% to 3.17%) in comparison to SPI and casein, exceeding the requirement by FAO/WHO/UNU (1985) for all groups including infants. Overall, CPI is an excellent source of arginine, glutamine, and histidine. The quality of proteins from the Brassica genus, as demonstrated by Sarwar and others (1984) is better than other plant proteins such as pea and wheat proteins. CPI (*B. napus*, cv. Tower) was also reported to have protein efficiency ratio (PER) of 2.64, exceeding PER of soybean meal that is only 2.19 (Delisle and others 1984).

There is little difference in amino acid composition between canola meal and CPI (Table 2). Rubin and others (1990) suggest that there was no loss of amino acid during the processing of canola meal; several redistributions among fractions may be possible as amino acid profile of CPI from SHMP extraction was only slightly different from its starting canola meal. However, there was a big reduction in the cysteine content of the CPI prepared by NaOH extraction. Tzeng and others (1988a) explained that hydrolysis and the degradation of some amino acid might have occurred due to the high pH and long-processing time. Genetic and environmental (geographical) differences have also been found to affect amino acid composition of canola seeds (Uppstrom 1995).

### Molecular weight

#### Canola meal

Protein profiles of meals from different Brassica species such as of *B.* napus were similar to *B. rapa* in nonreducing conditions with molecular weight of the polypeptides ranging from 12 to 80 kDa (Aluko and McIntosh 2001). They also reported that polypeptides of molecular weight 16, 18, 30, and 53 kDa were the 4 major polypeptides in the Brassica oilseeds studied, which accounted for over 55% of the total polypeptide composition of the canola meals. Molecular weight of the polypeptides in *B. juncea* meal also ranged from 2 to 80 kDa (Aluko and McIntosh 2005). While similar to the molecular weight range of proteins in *B. napus* and *B. rapa* meals, it is different to the protein profile of *Sinapis alba*. This is consistent with the findings by Rao and Rao (1981). Generally, comparison of the 4 oilseed varieties showed that *S. alba* meal contained more protein bands than either *B. juncea*, *B. rapa*, or *B. napus* in nonreducing conditions.

Protein profiles of the canola oilseeds in the presence of 2-mercaptoethanol (ME) show that the intensity of the major protein bands of *B. napus*, *B. rapa, and B. juncea* were reduced as a consequence of the disassociation of the disulfide linkages and breakdown of the polypeptides under reducing conditions (Aluko and McIntosh 2001; Aluko and others 2005). Concurrent with this was the appearance of additional protein bands, such as bands with molecular weight of 11 and 13 kDa in reduced protein profile of *B. napus* and *B. rapa* (Aluko and McIntosh 2001), consistent with findings of Venkatesh and Rao (1988). Polypeptide of 63 kDa molecular weight that was present in the protein profile of reduced *S. alba* meal was the major difference from the polypeptide profiles of Brassica oilseeds, as a result of dissociation of 135 kDa polypeptide that was available only in *S. alba* meal. The polypeptide profile of *S. alba* obtained under the reducing condition was consistent with other published results (Fischer and Schopfer 1988).

#### Canola protein isolates

Molecular weight analysis on CPI was recently conducted by Wu and Muir (2008). They reported that CPI (unknown species) showed 8 major bands with molecular weights ranging from 14 to 59 kDa, and at least 6 additional minor bands. The polypeptide band with molecular weight of 14 kDa was recognized as 2S albumin (napin) by comparing it to the protein profile of 2S albumin fraction that was separated and purified in the same study, this major band accounting for 25.3% of CPI. This was in agreement with the 13.4% to 46.1% range reported for napin of CPI (Schwenke 1994). Similar protein profile for purified napin was also reported by Krzyzaniak and others (1998). The band in CPI protein profile with a molecular weight of 27.5 kDa was probably a dimer of napin. This dimer and napin were dissociated under reducing conditions and resulted in the appearance of an additional band with 9.5 kDa molecular weight, indicating the presence of disulfide bonds in napin fractions of CPI.

The band with molecular weight of 59 kDa disappeared under reducing conditions; at the same time, additional band with molecular weight of 30.5 kDa appeared. Again, by comparing this with the reduced and nonreduced protein profile of cruciferin (the other major protein in canola/rapeseed), Wu and Muir (2008) suggested that this fraction was the dissociated polypeptide chain of the 59 kDa polypeptide that composed of 2 30.5 kDa polypeptides linked by disulfide bonds. Furthermore, polypeptide bands with molecular weight of 29.5, 44, 47.5, and 50 kDa also disappeared in the presence of reducing agent that broke up the disulfide linkages of the respective molecules. Certain bands such as polypeptides with molecular weight of 20 to 24 kDa, however, remained in the presence of reducing agent, indicating that these polypeptides are not stabilized by disulfide bonds. This suggests that the protein molecules of cruciferin are more complex; presumably, they are supported by not only disulfide bonds but also by noncovalent interactions (Dalgalarrondo and others 1986; Schwenke 1994).

### Molecular structure

The rapeseed protein is mainly composed of 12S cruciferin and 2S napin protein fractions as shown in the SDS polyacrylamide gel electrophoresis (PAGE) profiles. Both of these fractions are the characteristic storage proteins for seeds of the Brassica family that determine the nutritive and functional properties of the total rapeseed protein (Prakash and Rao 1986; Schwenke 1990). Napin has a high content of α-helical structure (40% to 46%) and a low content of β-sheet conformation (12%) in the secondary structure (Schwenke 1994). Cruciferin, on the other hand, has low content of α-helical structure (10%) and a high content of β-sheet conformation (50%) (Zirwer and others 1985).

A significant amount of research studies have been conducted on plant protein secondary structures. Comparatively, there was little information in literature on CPI secondary or tertiary structures, especially the influence of food-typical environmental conditions on these structures. Although a review of the structural and physiochemical properties of the rapeseed proteins was presented by Schwenke (1994), the review focused only on the major storage proteins of the rapeseed—napin and cruciferin in general, rather than on protein fractions that have different solubilities. Besides, the information on CPI molecular structure provided was insufficient in explaining its characteristics and food functional properties, suggesting that more research is definitely required in this area.

### pI of protein fractions

pI is the pH where protein has the lowest solubility. This information could be crucial in determining the utilization of a protein, especially in food processing. However, current literature mostly focused and discussed the pI of canola proteins in relation to the extraction procedures as shown in section 3, not in terms of molecular structure or food functionality. Besides, a systematic analysis of canola proteins using methods such as isoelectric focusing is currently lacking. More studies on the influence of environmental conditions commonly encountered in food systems such as pH and ionic strength, on canola proteins characteristics, and ultimately their functional properties are thus required.

### Solubility

Meals of various oilseed types show differences in solubility that may be variety specific. The process of oil extraction generally reduces the overall protein solubility (PS) (Pedroche and others 2004). Solubility of *B. napus* meal was 64.7% to 66.4% at pH 7, higher than solubility of meals from *B. rapa*, *B. juncea,* and *S. alba* that were 56.4% to 59.9%, 55.1%, 42.3% to 52.6%, respectively, at the same pH 7 (Aluko and McIntosh 2001; Aluko and others 2005). Another Brassica oilseed meal, *B. carinata*, had solubility of less than 40% at pH 7. Defatted soybean flour proteins were comparatively more soluble than those reported for defatted Brassica oilseed flours (Aluko and others 2005). Solubility of defatted soybean flour (67.7%) was found to be significantly higher than solubility of *B. juncea* and *S. alba* meals at pH 7.

As expected, solubility of CPI or original meal depends on the pH of solution. Pedroche and others (2004) carried out a detailed study on the solubility of *B. carinata* CPI and its meal at different pHs. In alkaline pH, solubility of protein isolates was higher; in acidic pH, solubility of meal was higher. Lower solubility of the meals at alkaline pH compared to CPI could be due to the fact that the meal contained other components that had low solubility. In addition, the heterogeneous nature of the meal may facilitate interaction between proteins and other components that can modify the net charge and hydrophobicity of protein thus affecting PS. Higher solubility of meal at acidic pH compared to CPI was explained by the fact that proteins soluble at low pH were lost during the preparation of CPI. As discussed earlier, CPI is frequently prepared from defatted meals by solubilization of proteins in alkaline media and precipitation at the acidic pI. It must be noted, however, that this study refers only to the precipitated proteins, not including the nonprecipitated (soluble) proteins that were collected in certain studies and been shown to have much better solubility (Yoshie-Stark and others 2008).

The ultrafiltered protein isolates had relatively higher solubility than precipitated CPI. According to Yoshie-Stark and others (2008), ultrafiltered CPI had a PS of 52.5% to 97.2% in the range pH 3 to 9, and greater than 90% at pH 5 to 9, in comparison to acid-precipitated protein isolate that was not solubilized at pH 3 and 4. The isolate, however, partially solubilized at pH 5 to 9 with solubility ranging from 21.3% to 26.4%. Solubility of ultrafiltered protein isolates was considerably higher if compared to solubility of commercial soy protein that was 62% at pH 10 (Lopez and others 2003). These conclusions should be treated with caution because solubility analysis method used by Aluko and others (2005) and Pedroche and others (2004) was slightly different from that of Yoshie-Stark and others (2008). High PS has been suggested as a critical factor that contributes to the functional properties of seed proteins such as emulsifying, foaming, and gelling properties (Kinsella and others 1985). The effect of PS on food functional properties will be discussed in more details in section 5.

### Hydrophobicity

Surface hydrophobicity of protein is often used to evaluate protein functionality. Hydrophobicity has been studied on plant proteins, for example, SPI (Jiang and others 2009), rice flour protein (Ju and others 2001), and hemp seed proteins (Tang and others 2009). However, there is very little information regarding hydrophobicity of CPI or the changes induced in aqueous environment, solvents, and proteolytic enzymes.

Uruakpa and Arntfield (2006a) reported that surface hydrophobicity of CPI was affected by the presence of a hydrocolloid (guar gum, κ-carrageenan) that generally increased the hydrophobicity of CPI. This could possibly be due to the interaction between CPI and the hydrocolloid that enhanced protein unfolding, thus exposing the buried hydrophobic amino acid residues. More research is needed in this area as it is important to have a better knowledge of how hydrophobicity of canola protein fractions affect their functional properties in food systems especially emulsification and fat/oil absorption properties.

### Thermal properties

Heating causes denaturation of protein as a result from the disruption of bonds that are involved in the formation and maintenance of the protein structure (Stanley and Yada 1994). The temperature needed and the extent of these changes were determined by the thermal stability of the protein, which can be studied from the endothermic peaks of their differential scanning calorimetry (DSC) profiles. The thermal stability of CPI, according to Wu and Muir (2008), was affected by a large number of factors, including protein structure, amino acid composition, binding of metals and other prosthetic groups, intramolecular interactions, protein–protein contacts, linkages, and environmental factors.

CPI shows 2 overlapping endothermic peak denaturation temperatures (T_d_) at 84 and 102 °C (Wu and Muir 2008). These 2 parallel transition peaks were contributed by its 2 major component proteins, cruciferin, and napin. Purified cruciferin and napin were shown to have higher T_d_ (91 and 110 °C, respectively) in comparison to those of the whole CPI. This could be due to the presence of nonprotein and other protein components in CPI that affect the thermal stability of proteins (Marcone and others 1998). As shown by Arntfield and Murray (1981), both the T_d_ and ethalpy of denaturation (ΔH) values were very similar to the proteins from other leguminous plant sources, such as soybean and faba bean.

Addition of β-ME, a reducing agent breaks the disulfide bond of cystinyl residues to sulfhydryl groups, decreased the thermal stability of cruciferin; T_d_ was significantly reduced from 91 to 76 °C (Wu and Muir 2008). The heat flow into the protein, defined by ΔH in the thermal denaturation process of cruciferin, was however not affected by ME. The authors suggest that noncovalent links are possibly more important in stabilizing the protein conformation of cruciferin than disulfide bonds. These observations were in agreement with the SDS PAGE profiles, in which adding ME changed only part of the polypeptide composition of cruciferin. The relatively high T_d_ value of napin indicates the high thermal stability of napin in comparison to cruciferin. This suggests that, unlike cruciferin, polypeptide chains of napin are mainly held together by disulfide bridges (Schwenke 1994) that are important in stabilizing the protein conformation of napin.

Overall, the available literature on canola protein characteristics shows that it is suitable for human consumption. The amino acid profile is comparable to that of proteins obtained from other sources such as soy and milk, and measures well against international dietary requirements, especially for young people and adults. Although numerous studies have been carried out on molecular weight profile of canola protein, there is limited literature that relates these findings to its functionalities. In comparison to other plant proteins, information on physicochemical properties of canola proteins, such as molecular structure, pI, and hydrophobicity, is still limited and thus, more studies are necessary.

## Food Functional Properties

Functional properties of proteins have been largely classified into 3 groups including (i) those related with hydration mechanisms such as water holding capacity and solublity, (ii) those related with structure and rheology such as thickening, viscosity, and gelation, and (iii) those related to protein surface such as foaming and emulsification (Damodaran 1997). In this review, however, based on the relative amount of information available about canola or rapeseed meals and proteins, their functional properties will be classified largely into 3 groups: emulsifying, foaming, and gelling.

### Emulsifying properties of canola meal

Proteins are an important group of emulsifying agents used in food. Proteins reduce the oil-water interfacial tension and thus facilitate the formation of emulsions as well as stabilize the oil droplets against coalescence (Kinsella 1982). During the process of emulsification, proteins with satisfactory emulsifying properties are able to adsorb rapidly at the newly created oil-water interfaces, followed by structural change and rearrangement at the oil-water interface, and subsequently the formation of a cohesive film with viscoelastic properties due to intermolecular interactions (Damodaran 1989). Many physicochemical factors are involved in this formation, stability, and textural properties of emulsions (Khattab and Arntfield 2009).

There are numerous studies on emulsifying properties of canola meals and protein isolates and these properties are commonly described by a few different terminologies. For example, emulsion activity index (EAI) and emulsifying capacity (EC) both of which indicate the ability of protein to form emulsion. EAI is a measure of available interfacial area that can be coated by the surfactant, for example, proteins, as explained by Pearce and Kinsella (1978). It is calculated from the turbidity of emulsion (T), volume fraction of dispersed phase (Ø), and the weight of protein per unit volume of aqueous phase before the emulsion is formed (C), using the formula EAI = 2T/ØC. In comparison to EAI, EC is a more straightforward indication determined by the volume of oil emulsified per gram meal (Khattab and Arntfield 2009) or per gram protein isolate (Yoshie-Stark and others 2008). Emulsion stability (ES), on the other hand, is measured by the percentage of volume of the emulsified layer after 30 min stand at room temperature compared to the initial volume of emulsion (Aluko and McIntosh 2001).

The EAI of *B. napus* and *B. rapa* canola meals were not significantly different from each other (Aluko and McIntosh 2001); this is in agreement with the findings from an earlier study by Naczk and others (1985). A difference in PS of these 2 meal varieties was reported, which indicates that emulsion formation was apparently not affected by PS. This, however, contradicts the finding by Kinsella and others (1985) who reported that increased PS led to increased ease of emulsion formation.

*Brassica juncea* meal had better emulsion forming ability compared to *B. napus* and *B. rapa*. EAI of *B. juncea* meal was also significantly higher when compared to *S. alba* meal (Aluko and others 2005). Increased EAI is related to lower molecular weights and better interfacial properties of protein molecules at the oil-water interface (Halling 1981). *Sinapis alba* meal has higher levels of high molecular mass polypeptides (50, 55, and 135 kDa) compared to Brassica oilseeds meal (Aluko and others 2005), which explains its lower EAI. Higher level of high molecular mass polypeptides denotes the presence of higher level of disulfide bonds, which could also have reduced the overall structural flexibility and interfacial property of the *S. alba* proteins.

In terms of ES, *B. napus* (cv.YN94-669) meal formed emulsion with a significantly lower ES compared to *B. rapa* meal, signifying that its proteins did not interact effectively at the interface to form a strong interfacial membrane (Aluko and McIntosh 2001). This has also been explained in terms of PS; *B. napus* cv. YN94-669 meal had the highest PS, suggesting that protein–protein interactions were less than protein-water interactions; hence, this meal formed the weakest interfacial membrane with least ES (Aluko and McIntosh 2001). This is consistent with the findings by Halling (1981) who suggested that strong protein–protein interactions at the oil-water interface was required for increased ES. On the other hand, a recent study by Khattab and Arntfield (2009) gave a different conclusion whereby it was established that high PS was required to achieve higher ES as well as better EAI. Despite the differences in the overall emulsifying properties (EAI and ES) of Brassica species meals studied by Aluko and his group, SDS PAGE showed similarities in polypeptide composition of the 4 seed types, indicating possible differences in protein structure or conformation (Aluko and McIntosh 2001) or possibly due to nonprotein components in the meals (Aluko and others 2005).

Soybean flour, as reported by Aluko and McIntosh (2004) and Aluko and others (2005), has better emulsifying properties (higher EAI and ES) than other reported Brassica oilseed meals. Nevertheless, there are few research studies (Ghodsvali and others 2005; Khattab and Arntfield 2009) that suggest that canola meals do possess better emulsifying properties. Although Ghodsvali and others (2005) studied the ability of proteins to form emulsion as EC instead of EAI, the results still show that canola meals (*B. napus*, cv. Quantum, PF, Hyola) have better emulsifying activity than the commercially produced soybean meal. Khattab and Arntfield (2009) also had similar findings; they reported that canola meal (*B. napus* cv. FortuneRR) was superior to soybean meals in its emulsifying properties. Thus, it is apparent that the emulsifying properties of canola meal, in comparison to soybean meal are dependent on the type of the canola meal and possibly the extraction and analytical methods.

Heat treatment was found to significantly reduce the EC and ES of canola meal (Khattab and Arntfield 2009). Moist heat treatment such as boiling or industrial desolventizing process during the canola oil extraction was found to have greater effect than dry heat treatment such as roasting. The lower EC and ES of heat-treated meals could be due to the denaturation of proteins and reduced nitrogen solubility.

ES by measuring the changes in particle size average and distribution is probably the most direct way of determining emulsification efficacy (Agboola and others 2007), although this type of analysis is yet to be meaningfully applied to the functionality of canola proteins. The application of this method to understand emulsion properties in a systematic way should assist in resolving some of the conflicting results outlined above.

### Emulsifying properties of CPI

According to Aluko and McIntosh (2001), emulsifying properties of acid-precipitated protein isolates (*B. napus* and *B. rapa*) were cultivar specific. Aluko and others (2005) reported that other acid-precipitated protein isolate from Brassica oilseeds such as *B. juncea* was found to have better emulsifying properties than either *B. napus* or *B. rapa* isolates. Relatively, acid-precipitated protein isolates form emulsions with higher stability, but calcium-precipitated protein isolates show higher capability to form emulsions.

Pedroche and others (2004) studied the effect of extraction pH on the emulsifying properties of acid-precipitated protein isolates. They found that protein isolates of *B. carinata* extracted at alkaline pH (either pH 10, 11, or 12) have lower emulsifying properties than its meal. As the extraction pH increased from pH 10 to 12, the emulsifying properties (both EC and ES) decreased. This is in contrast with the results reported by Aluko and McIntosh (2001), where the ES of acid-precipitated isolates was higher than that of its meal. This could be due to the differences in cultivars and extraction methodology as Pedroche and others (2004) used higher concentration of NaOH, longer extraction time, and precipitated the protein twice at both pH 3.5 and 5.0. Furthermore, the defatting process of the meal also had great effect on the emulsifying properties as well as other protein properties (Vioque and others 2000). These results call for a more systematic and comprehensive study on these important functional properties of canola meal proteins.

Ultrafiltered protein isolate (*B. napus* var. Express) has EC higher than that of whole egg (Yoshie-Stark and Wasche 2004), soy (Gao and others 2001), and many other plant proteins such as lupin (El-Adawy and others 2001), mung bean (El-Adawy 2000), pea (Gao and others 2001), and sesame (Khalid and others 2003). This suggests that ultrafiltered protein isolates have considerable emulsifying properties and may be better than the alkali-extracted isolates, most probably as a result of better overall PS.

Interactions of polysaccharides with CPI have been known to improve emulsifying properties. Uruakpa and Arntfield (2005b) found that the emulsifying properties of CPI were greatly improved by the addition of κ-carrageenan or guar gum. Different ideal pHs were required for interaction between CPI and different types of polysaccharides with pH 6 being the optimum pH for CPI-κ-carrageenan emulsion and pH 10 being the optimum pH for CPI-guar gum emulsion. Protein modification by hydrolysis is another common method for improving the solubility and hence emulsifying and other functional properties of proteins. This should be a valid means to explore for CPIs that are known to possess poor solubility, especially at neutral pHs.

### Foaming properties of canola meal and protein isolates

Foams are 2 phase systems composed of air bubbles surrounded by a continuous liquid lamellar phase (Sanchez-Vioque and others 2001). Foams can be formed and stabilized by either proteins or surfactants. Literature shows that canola proteins as foaming agents have been studied mainly in terms of foaming capacity (FC) and foam stability (FS). FC is related to the readiness of proteins to bind to the air-water interface to form foam particles, whereas FS is related to the protein–protein interactions that form strong interfacial membranes that stabilized the foam particles (Kinsella 1981). According to Aluko and McIntosh (2001), foaming properties of *B. juncea* meal were better than those of *S. alba* meal. In fact, the FC and foaming stability were even better than the results obtained for soybean flour. *Brassica napus* meal, in comparison, showed foaming properties that are significantly better than those from *B. rapa* meal and comparable to those of soybean flour. This is consistent with the findings from more recent studies by Ghodsvali and others (2005) and Khattab and Arntfield (2009). Even though the methods in analyzing FC were different, both studies consistently showed that the FC of canola meal was relatively higher than that of soybean meal.

The foaming properties of meals were better than its acid-precipitated or calcium-precipitated protein isolates. The process for preparing protein isolates reduced FC of *B. napus*, *B. rapa*, *B. juncea*, and *S. alba* meals consistently (Aluko and McIntosh 2001; Aluko and others 2005). The FS of the meals were also lower than those of protein isolates. This is in agreement with Pedroche and others (2004) who found that FC and FS of acid-precipitated protein isolates decreased if compared to the foaming properties of its meal. This could be due to the denaturation of proteins at high pH during the process of preparing the protein isolates. The study also found that acid-precipitated protein isolate (*B. carinata*) extracted at both pH 11 and 12 have reduced FC and FS as compared to protein isolate extracted at pH 10.

Protein isolates with high FC does not necessarily produce foam with high FS. As shown by Aluko and McIntosh (2001), *B. napus* cv. YN94-669, at pH 7, has the highest FC and also the least FS when compared to other varieties tested, indicating that while the proteins were bound more readily to the air-water interface during the formation of foams, the protein–protein interactions were not sufficiently strong to form stable interfacial membranes.

Acid-precipitated protein isolate had better foaming properties than the calcium-precipitated protein isolate generally (Aluko and McIntosh 2001). The foams formed with acid-precipitated protein isolate were more stable than those formed with calcium-precipitated protein isolate. For FC, mixed results were observed between acid-precipitated and calcium-precipitated protein isolates from different cultivars suggesting that this property may be specific to oilseed species. In comparison to SPI, foaming properties of SPI were better than those of either acid-precipitated or calcium-precipitated CPI.

Solubility of the canola meals or protein isolates is one of the important factors that contribute to their foaming properties. Meals with higher PS had better foaming properties (Aluko and McIntosh 2001). The ability of a protein to hold water in the film surrounding air particle is essential for FS (Kinsella and others 1985). These could possibly explain the higher FC and FS values of *B. napus* meal compared to *B. rapa* meal that possessed lower solubility.

Protein molecular size, presence of polyphenol, phytic acid, and heat treatment are among many other factors that contribute to the foaming properties of canola proteins. Adler-Nissen and Olsen (1979) showed that low molecular weight in proteins prevents the formation of stable foams. The presence of polyphenols, according to Sarker and others (1995), might be beneficial to foaming properties because polyphenols are involved in the stabilization of protein–protein complexes at the air-water interface. Phytic acid, on the other hand, interacts with proteins and form phytic acid-protein complexes that results in decreased PS (Schwenke and others 1987). Physical treatment such as heat processing was known to cause protein denaturation, thus reduced the FC and FS of canola proteins (Khattab and Arntfield 2009). Lin and others (1974) also had similar results, suggesting that native protein shows higher FS than a denatured one.

### Gelling properties of canola proteins

The gelling properties of canola proteins have been studied mostly in terms of least gelling concentration (LGC) (Gill and Tung 1978; Khattab and Arntfield 2009). Test tubes with various gelling concentrations were prepared by heating respective solutions or suspensions, and LGC was determined as the concentration in which the gel in the inverted test tubes did not slip. Other methods such as study of rheological properties (Pinterits and Arntfield 2007) or gel microstructure (Pinterits and Arntfield 2008) have been reported as well. Rapeseed flours, concentrates, and isolates were reported to possess poor gelation properties (Sosulski and others 1976). In contrast, Gill and Tung (1978) reported gelation of 12S glycoprotein fraction of rapeseed at protein concentrations as low as 4.5%, with measurable thickening at 1% protein. However, gelation mechanism and the bonds involved in gel formation and stability were not fully elucidated. They concluded that although some disulfide bonding was involved, ionic and hydrogen bonds were not likely to be major factors for cross-linking in the gel. More recent research by Khattab and Arntfield (2009) demonstrated that gelling properties of canola meal were relatively superior to those of soybean meal. Nevertheless, this was only a very general conclusion as some of the specific gelling properties of canola meal were not better than those of soybean, for example, the LGC of canola meal was higher than that of soybean, indicating poorer gelation characteristics.

Molecular size contributes to gelling properties of proteins as proteins with large molecular size were found to form more extensive networks by cross-linking in 3 dimensions, thus providing better gelling properties (Oakenfull and others 1997). Modification of protein structure, for example, by transglutaminase (TG) treatment, results in the cross-linking between polypeptides, thus leading to the formation of high molecular weight polymers. Hyun and Kang (1999) reported that canola proteins treated by TG are viable gelling agents. Pinterits and Arntfield (2007) suggested proteolysis as the pretreatment for cross-linking of proteins with TG. They found that proteolysis of canola proteins followed by TG treatment, produced canola proteins of significantly increased gelling properties, better than in nonhydrolysed proteins treated with TG. Limited proteolysis prior to TG treatment leads to partial unfolding of the protein structure, exposing buried lysine, and glutamine residues that were now available for cross-linking (Kang and others 1994).

Properties of gels produced from canola proteins can also be improved by the addition of polysaccharides. The inclusion of low levels of polysaccharides has been shown to improve gel properties in comparison to canola protein alone (Cai and Arntfield 1997). For example, the compatibility between CPI and κ-carrageenan was able to produce sufficient covalent linkages to form a gel when neither noncovalent interactions nor disulfide bonding were available (Uruakpa and Arntfield 2006b).

Contradictory findings have been reported in the literature with regard to canola protein functional properties. This could be due to the differences in the canola varieties, preparation procedures for the protein isolates, and methods of analyzing these functional properties. Apparently due to issues with currently available isolates, literature also shows that many studies in this area were focused on modification of canola protein, as well as its interactions with other food components such as polysaccharides, thus expanding the possibility of wider utilization of canola protein in human food.

## Conclusions

The potential for the utilization of canola meal proteins in food processing is supported by the fact that canola proteins are balanced in all essential amino acids, having a better amino acid profile than soybean protein isolates and comparing favorably with the amino acid requirements by FAO/WHO/UNU for both adults and children. Although antinutritional factors, color and the taste of the canola proteins are major obstacles for their use in human consumption, targeted extraction procedures should be able to overcome these problems.

Various methods for preparing CPI have been reviewed with the majority of these studies being based on alkaline extraction presumably due to high nitrogen yield. However, proteins extracted by alkali were not very suitable as food ingredients probably due to irreversible denaturation during the isolation process. As solubility is often considered to be a prerequisite for the performance of proteins in food applications, it is significant that protein isolates from alkaline extraction of canola meal have poor solubility at neutral pHs and poor technological functionalities. Meanwhile, there is evidence of significant amount of water- and salt-soluble proteins in Brassica species. Thus, a more comprehensive study is warranted that would be based on the utilization of these soluble fractions in order to provide a better understanding of the characteristics and functionality of canola proteins in food application.
